# Hemodynamic resilience and enhanced early recovery with fospropofol versus propofol in high-BMI patients undergoing surgery: a randomised controlled trial

**DOI:** 10.3389/fsurg.2026.1877622

**Published:** 2026-07-31

**Authors:** Cuiyuan Huang, Yue Song, Jia Li, Yanping Lu, Guangyi Lai, Zuofeng Wang, Shan Ou

**Affiliations:** 1 Department of Anaesthesiology, Chengdu Integrated TCM & Western Medicine Hospital, Chengdu, China; 2 Department of Anaesthesiology, Guangxi Medical University Cancer Hospital, Nanning, China; 3 Department of Oncology, Chengdu Seventh People's Hospital, Chengdu, China

**Keywords:** emergence, fospropofol, hemodynamic stability, high BMI, hypotension, pre-incision phase, propofol, surgical type

## Abstract

**Background:**

Patients with elevated body mass index (BMI) are particularly susceptible to propofol-induced hypotension during anesthesia induction. Fospropofol, a water-soluble prodrug of propofol, may confer hemodynamic advantages through gradual release kinetics, yet its profile in high-BMI patients remains incompletely characterized.

**Methods:**

This pre-specified secondary analysis of a randomised controlled trial (ChiCTR2500101725) enrolled 120 patients with BMI ≥28 kg/m² scheduled for elective surgery, randomised to fospropofol (FOS, *n* = 60) or propofol (PRO, *n* = 60). The primary endpoint of this analysis was hypotension incidence (any MAP <65 mmHg) during the pre-incision phase. Key secondary endpoints included hypotension area under the curve (AUC), MAP variability, emergence time, extubation time, and hospital length of stay.

**Results:**

Hypotension incidence was 30.0% (FOS) vs. 71.7% (PRO) [RR 2.39, 95% CI 1.57–3.63; *p* < 0.001; NNT 2.4 (i.e., one additional patient needs to be treated with fospropofol rather than propofol to prevent one case of hypotension)]. FOS patients had reduced hypotension burden (median AUC 0.0 vs. 29.5 mmHg·min; *p* < 0.001), lower MAP variability (8.7 ± 2.2% vs. 19.0 ± 7.4%; Cohen's d = 1.87), and higher MAP nadir (median 69.5 vs. 55.0 mmHg; *p* < 0.001). Emergence time was shorter with FOS (7.9 ± 2.7 vs. 10.7 ± 3.4 min; d = 0.91), as was extubation time (10.0 ± 2.7 vs. 12.7 ± 3.5 min; d = 0.86). A significant treatment × surgery interaction for extubation time (*p* = 0.042) indicated amplified benefit in abdominal procedures (*Δ* = −3.58 min vs. −1.07 min).

**Conclusions:**

Fospropofol demonstrated superior hemodynamic stability during the pre-incision phase and faster early recovery compared with propofol in high-BMI patients, particularly in abdominal surgical procedures.

**Clinical Trial Registration:**

https://www.chictr.org.cn/showproj.html?proj=253889, identifier ChiCTR2500101725 (registered 29 April 2025).

## Introduction

Induction of general anesthesia represents a period of heightened hemodynamic vulnerability, particularly in patients with elevated body mass index (BMI). Propofol, the most widely used intravenous induction agent, is associated with dose-dependent cardiovascular depression mediated by reduced systemic vascular resistance and myocardial contractility (1). In obese and overweight patients, the physiological consequences of propofol-induced hypotension are compounded by altered pharmacokinetics, increased blood volume, and reduced functional residual capacity, which collectively increase susceptibility to end-organ hypoperfusion (2, 3).

The clinical significance of intraoperative hypotension has been established in large observational studies. Even brief episodes of mean arterial pressure (MAP) below 65 mmHg are independently associated with acute kidney injury, myocardial injury, and increased mortality (4, 5). During the pre-incision phase specifically, the rapid transition from consciousness to general anesthesia produces abrupt vasodilation and sympathetic depression, making this interval the period of greatest hemodynamic lability (6). In high-BMI patients, who frequently present with hypertension, diabetes, and other cardiovascular comorbidities, the margin for hemodynamic tolerance is further narrowed (7).

Fospropofol disodium is a water-soluble phosphate ester prodrug of propofol that undergoes conversion by alkaline phosphatase to release propofol, phosphate, and formaldehyde (8). This enzymatic conversion introduces a pharmacokinetic delay that produces gradual increases in plasma propofol concentration, analogous to an endogenous target-controlled infusion (TCI) effect (9). In pharmacokinetic studies, fospropofol demonstrates a slower onset (approximately 4–8 min to peak effect) and more sustained plasma levels compared with direct propofol injection (10). While these properties suggest potential hemodynamic benefits, the existing evidence base consists primarily of studies in general surgical populations without specific focus on high-BMI patients or detailed pre-incision-phase hemodynamic profiling (11, 12).

This randomised controlled trial (ChiCTR2500101725) was designed to compare fospropofol with propofol in 120 patients with BMI ≥28 kg/m², incorporating detailed, minute-by-minute hemodynamic recordings during the pre-incision phase (1,354 time-series observations across 120 patients) and comprehensive recovery outcome data. Concurrently, the trial also evaluated metabolic endpoints. The present report focuses on the pre-specified hemodynamic and early recovery outcomes, providing a unique opportunity to characterize the hemodynamic resilience profile of fospropofol in a population at elevated risk for induction-related cardiovascular instability.

The primary objective of this study was to compare the incidence of hypotension (MAP <65 mmHg) during the pre-incision phase between fospropofol and propofol in high-BMI patients. Secondary objectives included characterization of hypotension burden (AUC), MAP variability, recovery times, and hospital length of stay, with pre-specified subgroup analysis by surgical type (abdominal vs. non-abdominal). We hypothesised that fospropofol would reduce hypotension incidence and that this hemodynamic advantage would translate into accelerated early recovery, with differential effects based on surgical category.

## Methods

### Study design and ethical approval

This prospective, randomised, single-blind, parallel-group controlled trial was registered in the Chinese Clinical Trial Registry (ChiCTR2500101725). The trial investigated hemodynamic and early recovery outcomes of fospropofol versus propofol in high-BMI patients, alongside metabolic endpoint evaluation. The study was approved by the institutional ethics committee, and written informed consent was obtained from all participants. The trial was originally powered for metabolic endpoints (ALP change at 24 h); hemodynamic and early recovery outcomes were pre-specified secondary endpoints. *post-hoc* power analysis confirmed 99.7% power for the observed hypotension incidence difference (Cohen's h = 0.86; *α* = 0.05, two-sided; *n* = 60 per group). The original trial protocol and statistical analysis plan are provided in Supplementary Data Sheet 1. Given the physical differences between fospropofol (clear aqueous solution) and propofol (white lipid emulsion), a double-blind design was not feasible. The following blinding measures were implemented: (1) Patients were blinded to group allocation throughout the study. (2) The outcome assessors responsible for recording emergence time, extubation time, and hemodynamic data were not involved in anaesthesia administration and were blinded to group assignment where feasible. (3) The attending anaesthesiologist who administered the study drug was necessarily unblinded due to the visual differences between formulations. (4) Data analysts (YS, JL) were blinded to group allocation during statistical analysis, with groups coded as A and B until the analysis plan was finalised.

### Participants

**Inclusion criteria:**One hundred twenty adult patients (aged 18–60 years) with BMI ≥28 kg/m², classified as American Society of Anesthesiologists (ASA) physical status I–III, scheduled for elective surgery under general anaesthesia were enrolled.

**Key exclusions:** Severe cardiovascular disease (ejection fraction <40%), known allergy to propofol or fospropofol, liver dysfunction (Child-Pugh class B or C), renal impairment (eGFR <60 mL/min/1.73 m²), and pregnancy or lactation.

### Randomisation and intervention

Patients were randomised 1:1 using a computer-generated sequence with block randomisation (block size 4) to receive either fospropofol (FOS group, *n* = 60) or propofol (PRO group, *n* = 60). FOS patients received fospropofol at a median induction dose of 7.2 mg/kg (IQR 6.7–7.6) and maintenance at 5.0 mg/kg/h (IQR 4.6–5.5). PRO patients received propofol at a median induction dose of 2.3 mg/kg (IQR 2.1–2.4) and maintenance at 7.0 mg/kg/h (IQR 5.2–9.9). Both groups received standardised opioid analgesia, muscle relaxation, and airway management per institutional protocol. Following induction, anesthesia depth was monitored using bispectral index (BIS) in all patients, with a target range of 40–60 throughout the procedure. The maintenance infusion rate (fospropofol or propofol) was titrated according to BIS feedback. If BIS exceeded 60, the infusion rate was increased; if BIS fell below 40, the rate was decreased. Sevoflurane was administered as an adjuvant, with end-tidal concentration adjusted to maintain BIS within the target range. Additional opioids or vasopressors were administered at the discretion of the attending anaesthesiologist according to standardized institutional protocols.

### Hemodynamic management protocol

A standardised hemodynamic management protocol was followed. When MAP decreased below 65 mmHg or by more than 30% from baseline, phenylephrine was administered as a bolus at the discretion of the attending anaesthesiologist. The timing and dose of all vasopressor administrations were recorded. Crystalloid and colloid fluid administration was recorded at each time point.

### Data collection and outcome measures

Intraoperative hemodynamic data (MAP, SBP, DBP, HR) were recorded at standardised intervals during the pre-incision phase, defined as the interval from anaesthesia initiation to surgical incision. MAP, SBP, DBP, and HR were recorded using non-invasive oscillometric monitoring at approximately 2-minute intervals throughout the pre-incision phase. The number of measurements per patient varied according to the duration of the pre-incision phase (median 11 measurements per patient, range 8–14). All recorded values were included in the analysis; values flagged as physiologically implausible (MAP < 20 mmHg or > 200 mmHg) underwent review against concurrent heart rate and SpO₂ recordings. No values were excluded. Hypotension AUC was calculated from raw MAP values without adjustment for vasopressor administration. The pre-incision phase spanned approximately 0–26 min relative to anaesthesia start time, yielding 1,354 individual time-series measurements across all participants. The duration of the pre-incision phase varied according to the specific surgical procedure (median 23.0 min, IQR 18.0–26.5, range 15.0–30.0) and did not differ between groups (FOS 22.5 ± 4.8 vs. PRO 22.6 ± 4.7 min; *p* = 0.899). The primary endpoint (any MAP <65 mmHg) was assessed throughout the entire pre-incision period for each patient, regardless of duration. Sensitivity analyses restricted to the first 15 min of monitoring confirmed the robustness of the primary finding (FOS 28.3% vs. PRO 70.0%; *p* < 0.001).

**Primary endpoint of this analysis: hypotension incidence,** The proportion of patients with any MAP recording below 65 mmHg during the pre-incision phase, based on established evidence linking MAP <65 mmHg with end-organ injury (4, 5).

**Key secondary endpoints:** (1) Hypotension burden, quantified as the area under the curve (AUC) of MAP deficit below 65 mmHg over time (mmHg·min), calculated using the trapezoidal method; (2) MAP coefficient of variation (CV); (3) MAP nadir; (4) MAP nadir as a percentage of baseline; (5) emergence time; (6) extubation time; (7) hospital length of stay.

### Statistical analysis

Categorical variables were compared using chi-square or Fisher's exact tests, with relative risk (RR) and 95% confidence intervals (CI). The original sample size of 120 patients (60 per group) was calculated for the metabolic primary endpoint (ALP change at 24 h). Based on an expected between-group difference of 12.5 U/L (SD 25 U/L) derived from pilot data, *α* = 0.05 (two-sided), and 80% power, 54 participants per group were required. This was inflated to 60 per group to allow for a 10% attrition rate. For the hemodynamic primary endpoint of this secondary analysis, a *post-hoc* power calculation was performed: with the observed hypotension incidence of 30.0% (FOS) versus 71.7% (PRO), Cohen's h = 0.86, the achieved power was 99.7% (*α* = 0.05, two-sided; *n* = 60 per group). The number needed to treat (NNT) was calculated from the absolute risk reduction. Continuous variables were assessed for normality using Shapiro–Wilk tests and for homogeneity of variance using Levene's test. Normally distributed data were compared using independent t-tests or Welch's *t*-tests; non-normally distributed data were analyzed using Mann–Whitney U tests. Effect sizes were quantified using Cohen's d for parametric and rank-biserial correlation for non-parametric comparisons.

Multivariable logistic regression was performed to identify independent predictors of hypotension, adjusting for treatment group, surgical type, baseline MAP, and BMI. A linear mixed-effects model was fitted with MAP as the dependent variable, incorporating fixed effects for time, treatment group, surgical type, and all interactions, with random intercepts for subjects. Treatment × surgical type interactions were tested using two-way ANOVA. Time-to-event analysis was performed using Kaplan–Meier estimation with log-rank testing.

Pre-specified subgroup analyses were conducted for BMI category (28–32 vs. > 32 kg/m²), surgical type, hypertension status, and diabetes status. Sensitivity analyses included ANCOVA adjusting for baseline MAP and BMI, an alternative hypotension definition (MAP decline >30% from baseline), and Holm-Bonferroni correction. All analyses were performed using Python 3.12 with scipy 1.14, statsmodels 0.14, and lifelines 0.29. Two-sided *p* < 0.05 was considered statistically significant.

## Results

### Baseline characteristics

**Figure 1:**
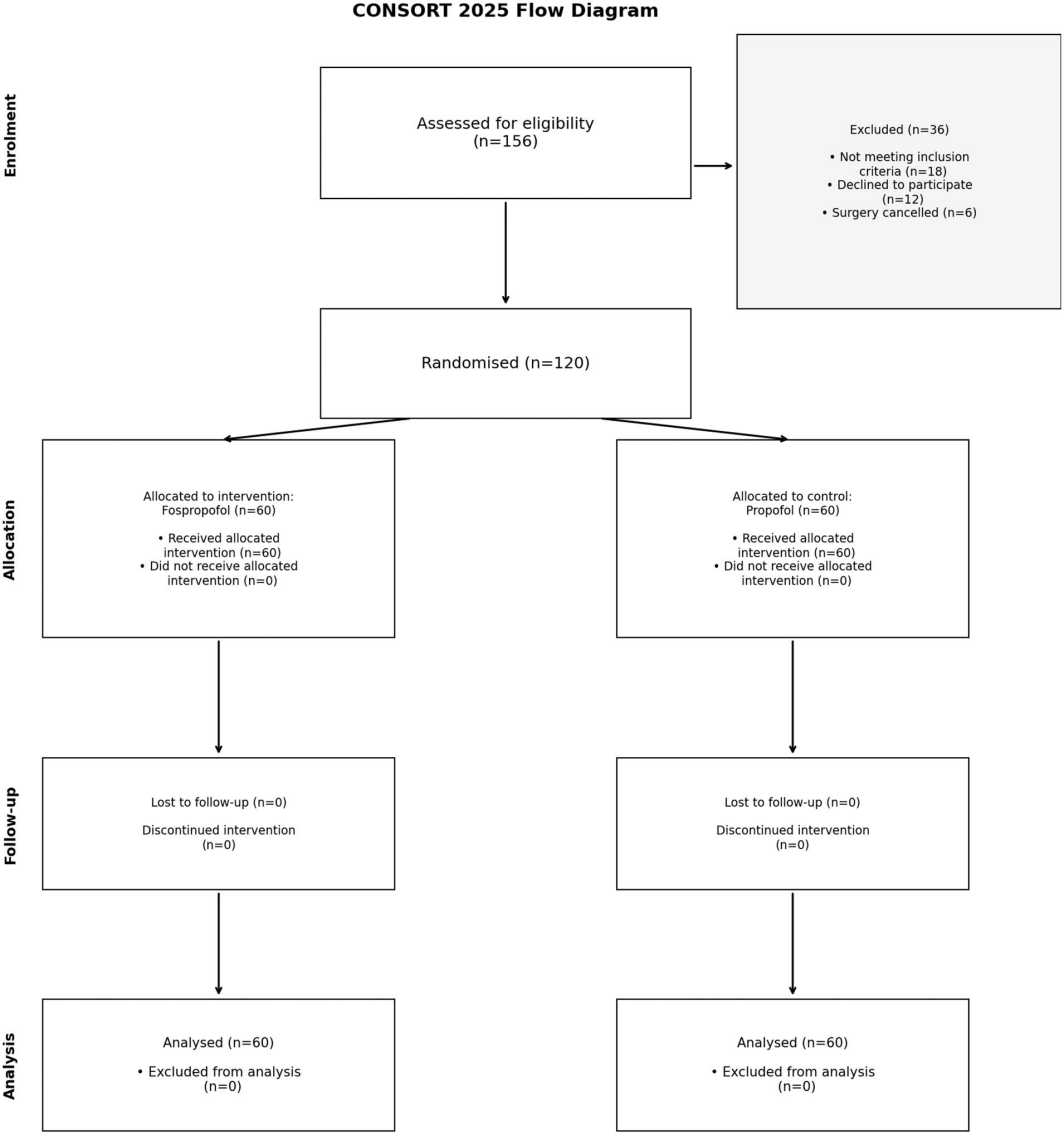
CONSORT flow diagram showing participant enrollment, randomisation, and follow-up. ITT = intention-to-treat. One hundred fifty-six patients were assessed for eligibility; 36 were excluded (18 did not meet inclusion criteria, 12 declined participation, 6 had scheduling conflicts), and 120 were randomised (60 per group). All randomised patients completed the study and were included in the analysis with no losses to follow-up.

One hundred twenty patients were included (60 per group), with no losses to follow-up (Figure 1). The two groups were well balanced with respect to demographic and clinical characteristics (Table 1). Median age was 40.8 years (IQR 35.8–54.4) in the FOS group and 46.9 years (IQR 37.8–54.2) in the PRO group. Median BMI was comparable (31.6 vs. 31.5 kg/m²). Baseline hemodynamic parameters were balanced, including SBP (136.1 ± 16.8 vs. 134.5 ± 14.2 mmHg), baseline MAP (98.3 vs. 100.2 mmHg), and heart rate (78.7 ± 12.2 vs. 78.5 ± 13.3 bpm). A modest imbalance was observed in ASA class III distribution (FOS 10.0% vs. PRO 20.0%; SMD = 0.280, *p* = 0.142).

**Table 1:** Baseline characteristics.

Variable	FOS (*n* = 60)	PRO (*n* = 60)	SMD	*p*-value
Age (years)	40.8 [35.8–54.4]	46.9 [37.8–54.2]	0.164	0.465
Female sex, *n* (%)	37 (61.7%)	32 (53.3%)	0.169	0.460
BMI (kg/m²)	31.6 [28.9–33.7]	31.5 [29.8–33.4]	0.170	0.390
ASA class III, *n* (%)	6 (10.0%)	12 (20.0%)	0.280	0.142
Hypertension, *n* (%)	24 (40.0%)	22 (36.7%)	0.069	0.851
Diabetes, *n* (%)	18 (30.0%)	15 (25.0%)	0.112	0.683
SBP (mmHg)	136.1 ± 16.8	134.5 ± 14.2	0.105	0.567
Baseline MAP (mmHg)	98.3 [94.9–104.1]	100.2 [94.5–105.7]	0.052	0.536
Heart rate (bpm)	78.7 ± 12.2	78.5 ± 13.3	0.016	0.932
Abdominal surgery, *n* (%)	43 (71.7%)	35 (58.3%)	0.282	0.180
Surgery duration (min)	116.0 [88.5–150.5]	112.0 [82.8–142.0]	0.124	0.503
Blood loss (mL)	190.5 [139.8–245.2]	200.5 [135.5–255.0]	0.017	0.877

### Primary outcome: hypotension incidence

Hypotension during the pre-incision phase occurred in 18/60 (30.0%) patients in the FOS group compared with 43/60 (71.7%) in the PRO group (*p* < 0.001; Table 2, Figure 3). The relative risk for propofol-associated hypotension was 2.39 (95% CI 1.57–3.63), and the NNT was 2.4 (95% CI 1.7–3.9), indicating that treating approximately two to three patients with propofol rather than fospropofol would result in one additional case of hypotension.

**Table 2:** Primary and secondary hemodynamic outcomes.

Outcome	FOS (*n* = 60)	PRO (*n* = 60)	Effect Size (95% CI)	*p*-value
Primary: Hypotension incidence	18/60 (30.0%)	43/60 (71.7%)	RR 2.39 (1.57–3.63)	<0.001
NNT	—	—	2.4 (1.7–3.9)	—
Hypotension AUC (mmHg·min)	0.0 [0.0, 2.5]	29.5 [0.0, 51.0]	r = 0.51	<0.001
MAP CV (%)	8.7 ± 2.2	19.0 ± 7.4	d = 1.87 (1.49–2.36)	<0.001
MAP nadir (mmHg)	69.5 [63.0, 80.0]	55.0 [50.0, 66.2]	r = 0.55	<0.001
MAP nadir (% baseline)	70.9 [63.8, 79.1]	55.6 [49.8, 67.9]	r = 0.52	<0.001
SBP CV (%)	8.2 ± 2.4	14.4 ± 4.8	d = 1.64 (1.29–2.07)	<0.001
HR CV (%)	15.3 ± 3.6	14.0 ± 3.0	d = 0.36	0.051

Multivariable logistic regression confirmed that propofol use was an independent predictor of hypotension (OR = 6.07; 95% CI 2.59–14.21; *p* < 0.001), after adjustment for surgical type, baseline MAP, BMI, and ASA physical status (Table 3). Abdominal surgery was associated with lower hypotension risk (OR = 0.35; 95% CI 0.14–0.90; *p* = 0.029). ASA class showed a trend toward lower hypotension risk (OR = 0.50; 95% CI 0.25–1.00; *p* = 0.051). The model demonstrated acceptable discrimination (AUC-ROC = 0.785) and calibration (Hosmer-Lemeshow *p* = 0.431).

**Table 3:** Multivariable logistic regression for hypotension (Hosmer-Lemeshow *p* = 0.431; AUC-ROC = 0.785).

Variable	OR	95% CI	*p*-value
Propofol (vs. Fospropofol)	6.07	2.59–14.21	<0.001
Abdominal surgery (vs. Non-abdominal)	0.35	0.14–0.90	0.029
Baseline MAP (per mmHg)	1.06	1.00–1.12	0.061
BMI (per kg/m²)	1.14	0.97–1.34	0.119
ASA class	0.50	0.25–1.00	0.051

### Secondary hemodynamic outcomes

**Figure 2:**
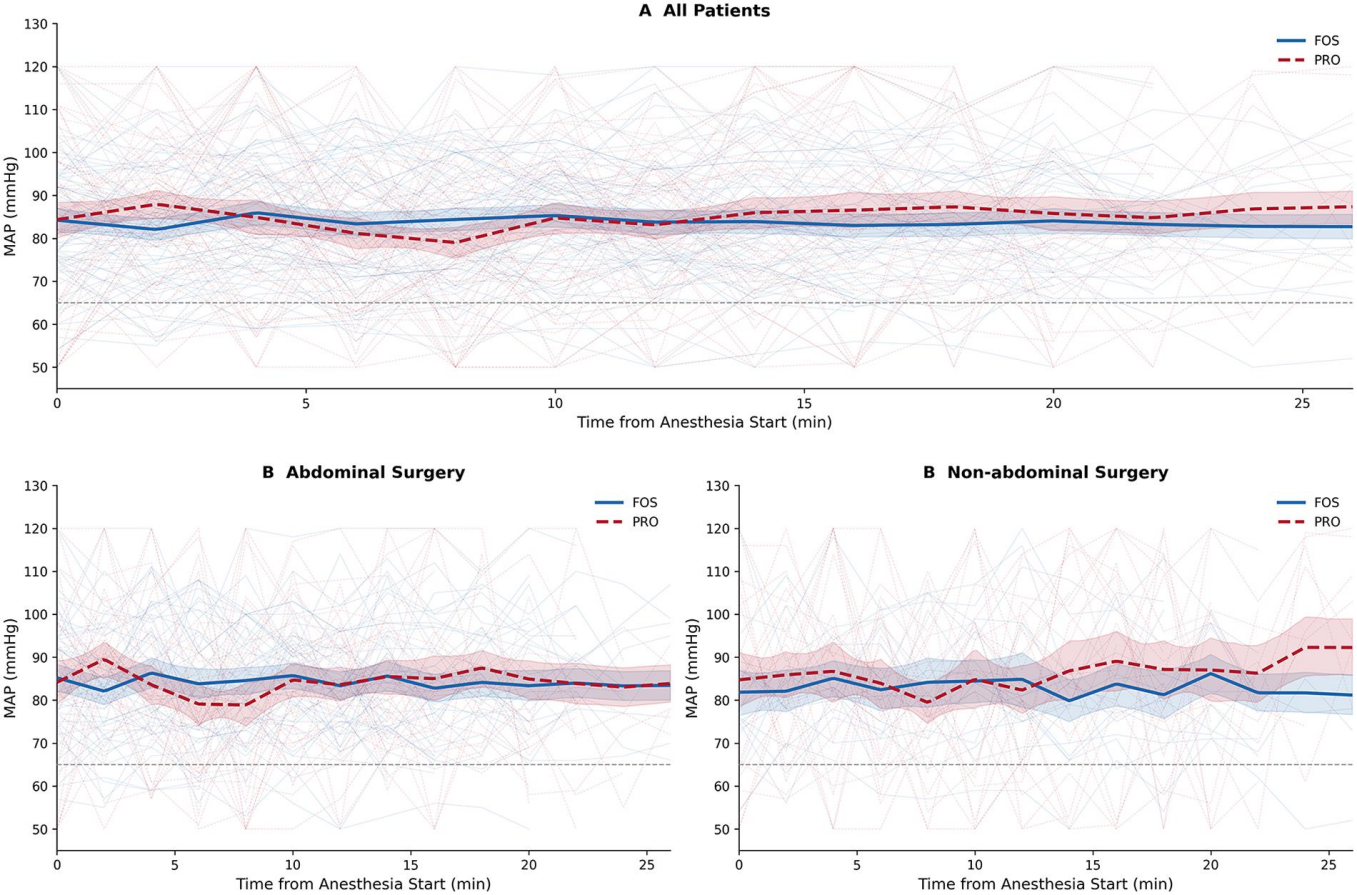
Mean arterial pressure (MAP) trajectories during the pre-incision phase. **(A)** Overall MAP trajectories for the fospropofol (FOS; blue) and propofol (PRO; red) groups. Spaghetti plots (*α* = 0.08) are overlaid with locally weighted smoothed mean curves and 95% confidence intervals. The dashed horizontal line represents MAP = 65 mmHg. **(B)** MAP trajectories stratified by surgical type.

**Figure 3:**
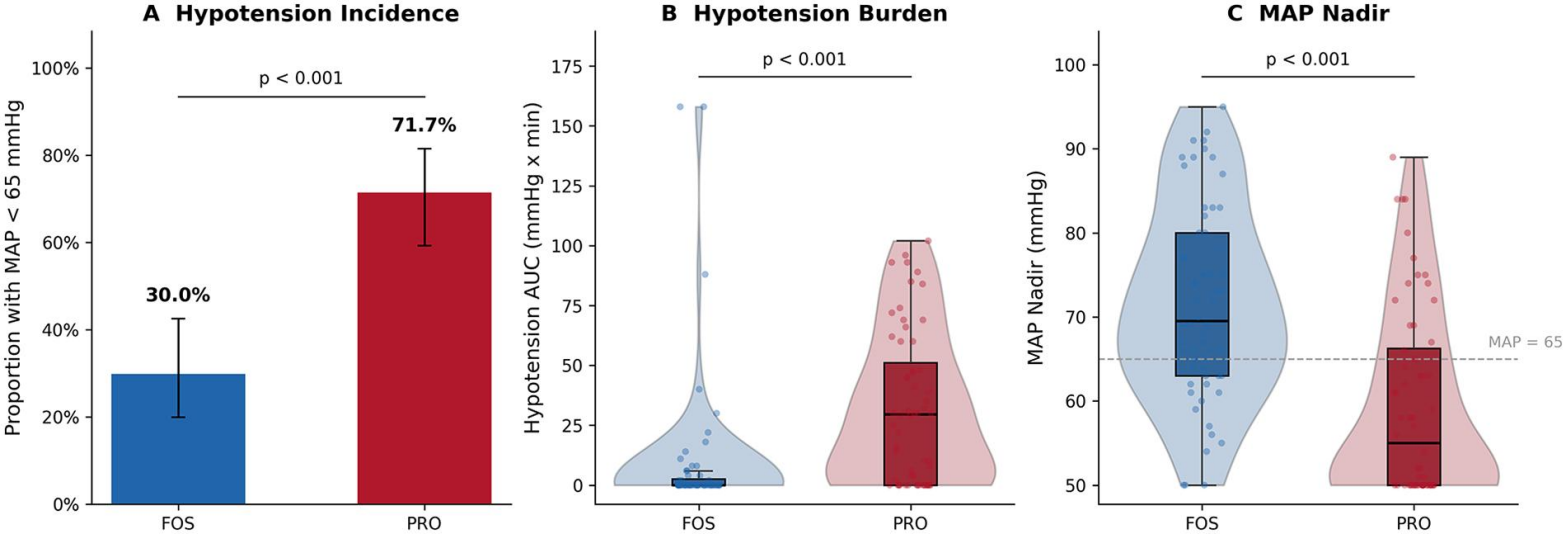
Hypotension burden during the pre-incision phase. **(A)** Hypotension incidence: proportion of patients with any MAP <65 mmHg with Wilson score 95% CI. **(B)** Hypotension AUC (mmHg·min) distributions as violin/box/strip plots. **(C)** MAP nadir distributions. The dashed line indicates MAP = 65 mmHg. FOS, blue; PRO, red.

All secondary hemodynamic endpoints significantly favored the FOS group (Table 2, Figures 2, 3). Hypotension burden (AUC below MAP 65 mmHg) was markedly lower in the FOS group (median 0.0, IQR 0.0–2.5 vs. 29.5, IQR 0.0–51.0 mmHg·min; *p* < 0.001; Figure 3B). MAP coefficient of variation was 8.7 ± 2.2% in the FOS group versus 19.0 ± 7.4% in the PRO group (Cohen's d = 1.87; *p* < 0.001), representing a nearly twofold increase in blood pressure variability with propofol. MAP nadir was significantly higher in the FOS group (median 69.5, IQR 63.0–80.0 vs. 55.0, IQR 50.0–66.2 mmHg; *p* < 0.001; Figure 3C). The MAP nadir as a percentage of baseline was 70.9% vs. 55.6% (median; *p* < 0.001).

SBP variability followed a similar pattern (FOS 8.2 ± 2.4% vs. PRO 14.4 ± 4.8%; Cohen's d = 1.64; *p* < 0.001). Heart rate variability did not differ significantly (FOS 15.3 ± 3.6% vs. PRO 14.0 ± 3.0%; *p* = 0.051), suggesting that the hemodynamic advantage of fospropofol is primarily mediated through vascular rather than chronotropic effects.

The linear mixed-effects model for longitudinal MAP trajectories revealed a trend toward a treatment × time interaction (*β* = 0.31 per minute; *p* = 0.094), indicating that the MAP divergence between groups tended to increase over the pre-incision period. The three-way interaction between treatment, time, and surgical type was not statistically significant (*β* = −0.42; *p* = 0.071).

### Anesthetic depth and concomitant medication

Under BIS-guided anesthesia management (target 40–60), anesthesia depth was comparable between groups. Sevoflurane end-tidal MAC values were equivalent (FOS 1.00 ± 0.04 vs. PRO 1.01 ± 0.04; *p* = 0.217), indicating similar volatile anesthetic requirements to maintain the target BIS range. Opioid exposure was consistent (fentanyl per interval: FOS 200.1 ± 20.1 mcg vs. PRO 200.7 ± 17.7 mcg; *p* = 0.865), as was muscle relaxant dosing (rocuronium per interval: FOS 59.4 ± 3.4 mg vs. PRO 59.4 ± 3.6 mg; *p* = 0.995). These data indicate that the observed hemodynamic differences cannot be attributed to differential anesthetic depth or adjuvant medication (Table 4).

**Table 4:** Anesthetic depth, concomitant medication, and intraoperative fluid management.

Variable	FOS (*n* = 60)	PRO (*n* = 60)	*p*-value
Anesthetic depth			
Sevoflurane MAC	1.00 ± 0.04	1.01 ± 0.04	0.217
Fentanyl per interval (mcg)	200.1 ± 20.1	200.7 ± 17.7	0.865
Rocuronium per interval (mg)	59.4 ± 3.4	59.4 ± 3.6	0.995
Maintenance rate (mg/kg/h)	5.0 ± 0.6	7.5 ± 2.4	<0.001
Vasopressor use			
Phenylephrine use, *n* (%)	60 (100.0)	58 (96.7)	0.154
Events per patient, median (IQR)	3 (2–4)	4 (2–5)	0.679
Cumulative phenylephrine dose (*μ*g)	119.4 ± 138.2	115.9 ± 107.1	0.687
Fluid management			
Crystalloid (mL)	1,018.1 ± 96.4	1,004.8 ± 80.7	0.418
Colloid (mL)	251.8 ± 34.8	250.0 ± 40.9	0.805
Estimated blood loss (mL)	173.0 ± 25.7	176.4 ± 21.0	0.436

Values are mean ± SD or median (IQR) per recording interval, except phenylephrine cumulative dose (per patient) and events per patient. MAC, minimum alveolar concentration. *P*-values from independent-samples t-tests or Mann–Whitney U tests as appropriate.

Vasopressor (phenylephrine) was administered to 60/60 (100.0%) patients in the FOS group and 58/60 (96.7%) in the PRO group (*p* = 0.154). The cumulative phenylephrine dose per patient was comparable (FOS 119.4 ± 138.2 μg vs. PRO 115.9 ± 107.1 μg; *p* = 0.687). Crystalloid volume was similar between groups (FOS 1,018.1 ± 96.4 mL vs. PRO 1,004.8 ± 80.7 mL; *p* = 0.418). Colloid administration was also comparable (FOS 251.8 ± 34.8 mL vs. PRO 250.0 ± 40.9 mL; *p* = 0.805). Estimated blood loss did not differ between groups (FOS 173.0 ± 25.7 mL vs. PRO 176.4 ± 21.0 mL; *p* = 0.436) (Table 4).

### Recovery outcomes

**Figure 4:**
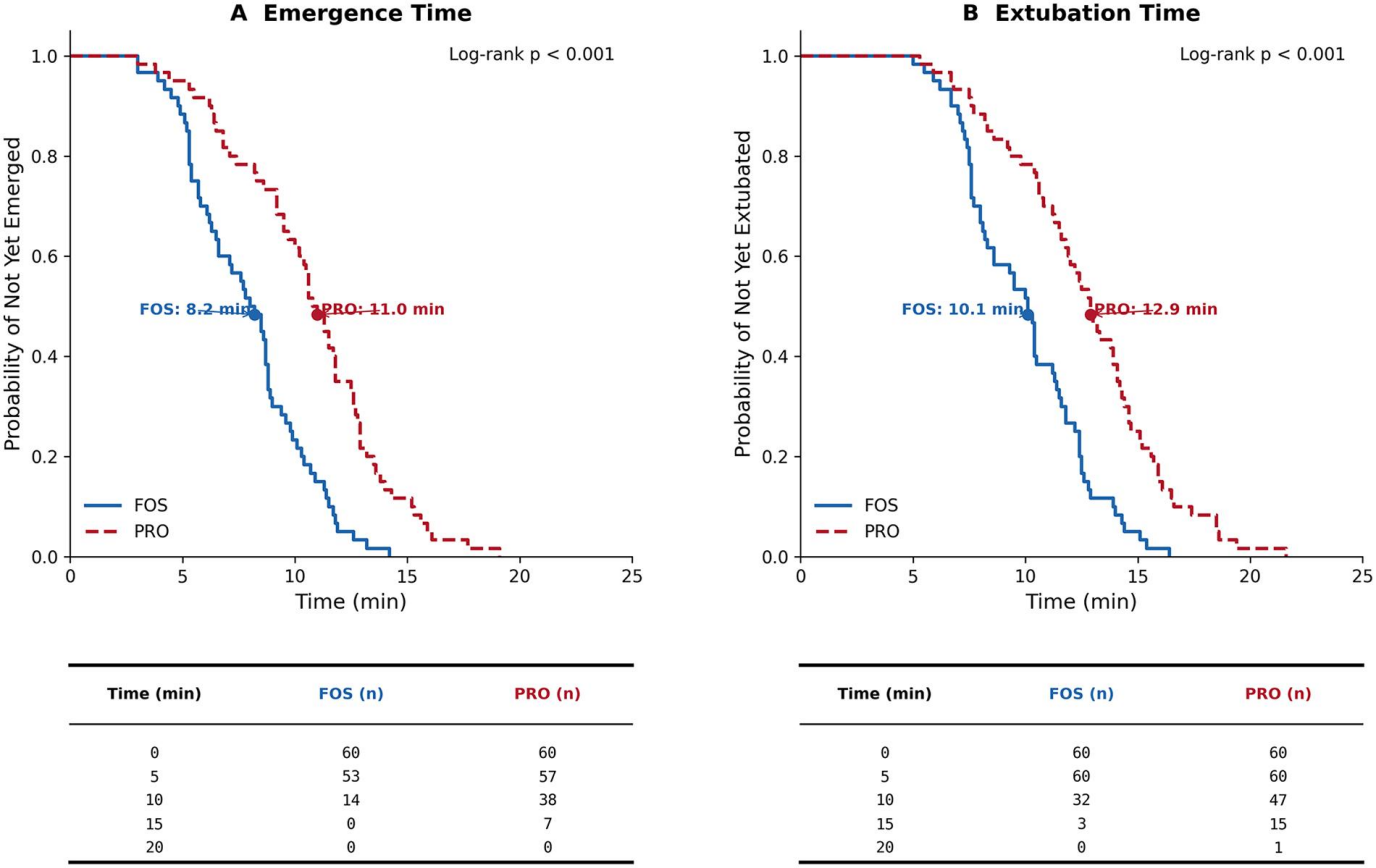
Kaplan–Meier curves for recovery time endpoints. **(A)** Time to emergence (eye opening on command) and **(B)** time to extubation. FOS group shown in blue, PRO group in red. Median times are annotated. At-risk tables are provided below each panel.

Emergence time was 2.8 min shorter in the FOS group (7.9 ± 2.7 vs. 10.7 ± 3.4 min; Cohen's d = 0.91; *p* < 0.001; Table 5). Extubation time was similarly reduced (10.0 ± 2.7 vs. 12.7 ± 3.5 min; Cohen's d = 0.86; *p* < 0.001). Kaplan–Meier analysis confirmed significantly faster time to emergence (median 8.2 vs. 11.0 min; log-rank *χ*² = 26.25; *p* < 0.001; Figure 4A) and extubation (median 10.1 vs. 12.9 min; log-rank *χ*² = 23.48; *p* < 0.001; Figure 4B).

**Table 5:** Recovery outcomes and treatment × surgery type interactions.

Outcome	FOS (*n* = 60)	PRO (*n* = 60)	Diff (95% CI)	p	Effect Size
Emergence (min)	7.9 ± 2.7	10.7 ± 3.4	−2.79 (−3.90, −1.67)	<0.001	d = 0.91
Extubation (min)	10.0 ± 2.7	12.7 ± 3.5	−2.72 (−3.86, −1.57)	<0.001	d = 0.86
LOS (days)	2.7 ± 1.0	3.2 ± 1.3	−0.56 (−0.98, −0.13)	0.011	d = 0.47
PONV 0–24 h	9/60 (15.0%)	11/60 (18.3%)	RR 0.82 (0.37–1.83)	0.624	—
Pain PACU VAS	5.0 [3.0, 7.0]	5.0 [3.0, 6.0]	—	0.565	r = −0.06
Pain 24 h VAS	3.0 [2.0, 4.0]	3.0 [2.0, 4.0]	—	0.708	r = 0.04
Satisfaction	8.0 [7.0, 9.0]	8.0 [7.8, 9.0]	—	0.140	r = 0.15
Emergence: interaction			F = 3.48	0.065	
Extubation: interaction			F = 4.21	0.042	
Emergence KM (log-rank)	median 8.2	median 11.0	*χ*² = 26.25	<0.001	
Extubation KM (log-rank)	median 10.1	median 12.9	χ² = 23.48	<0.001	

Hospital length of stay was shorter in the FOS group (2.7 ± 1.0 vs. 3.2 ± 1.3 days; Cohen's d = 0.47; *p* = 0.011). Postoperative nausea and vomiting (PONV) incidence was comparable (15.0% vs. 18.3%; RR 0.82; *p* = 0.624). Pain scores and patient satisfaction did not differ between groups (Table 5).

### Surgical type stratification

A significant treatment × surgical type interaction was identified for extubation time (F = 4.21; *p* = 0.042; Table 5). Among patients undergoing abdominal surgery (*n* = 78), the extubation time difference was 3.58 min (FOS 9.6 ± 2.5 vs. PRO 13.2 ± 3.7 min; *p* < 0.001; Cohen's d = 1.14), whereas in non-abdominal surgery (*n* = 42), the difference was 1.07 min (FOS 11.0 ± 3.1 vs. PRO 12.1 ± 3.2 min; *p* = 0.284; Cohen's d = 0.34).

A similar pattern was observed for emergence time, with an interaction *p*-value of 0.065, suggesting a trend toward greater FOS benefit in abdominal procedures (*Δ* = 3.57 vs. 1.36 min). Hypotension incidence was significantly lower with FOS in both surgical categories (abdominal: 27.9% vs. 60.0%, *p* = 0.006; non-abdominal: 35.3% vs. 88.0%, *p* < 0.001), with a non-significant interaction (*p* = 0.183).

### Subgroup and sensitivity analyses

**Figure 5:**
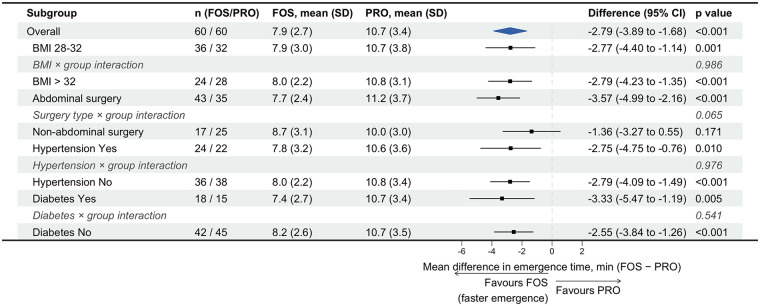
Forest plot of subgroup analyses for emergence time. Mean difference (FOS−PRO) in emergence time with 95% confidence intervals is shown for the overall estimate (summary diamond) and each subgroup. Subgroups include BMI category, surgical type, hypertension status, and diabetes status. Italic rows indicate interaction *p*-values. FOS, fospropofol; PRO, propofol.

Subgroup analyses across BMI category (BMI 28–32: *n* = 84; BMI >32: *n* = 36), hypertension status (yes: *n* = 42; no: *n* = 78), and diabetes status (yes: *n* = 18; no: *n* = 102) demonstrated consistent FOS advantages across all strata (Supplementary Table S1, Figure 5). No significant treatment × subgroup interactions were detected for emergence time (BMI: *p* = 0.986; hypertension: *p* = 0.976; diabetes: *p* = 0.541) or extubation time (BMI: *p* = 0.936; hypertension: *p* = 0.854; diabetes: *p* = 0.589).

All primary findings were robust across sensitivity analyses. ANCOVA adjusting for baseline MAP and BMI confirmed the FOS advantage for emergence time (adjusted mean difference −2.79 min; 95% CI −3.91 to −1.66; *p* < 0.001), extubation time (−2.72 min; 95% CI −3.88 to −1.57; *p* < 0.001), and LOS (−0.57 days; 95% CI −1.00 to −0.14; *p* = 0.010). Using an alternative hypotension definition (MAP decline >30% from baseline), the between-group difference remained significant (FOS 45.0% vs. PRO 78.3%; RR 1.74; 95% CI 1.28–2.37; *p* < 0.001). Holm-Bonferroni correction for six hemodynamic comparisons maintained significance for all variables (five with adjusted *p* < 0.001; HR variability adjusted *p* = 0.030).

## Discussion

This randomised controlled trial demonstrates that fospropofol provides substantially superior hemodynamic stability during the pre-incision phase in patients with BMI ≥28 kg/m², with clinically meaningful reductions in hypotension incidence (30.0% vs. 71.7%), hypotension burden, and blood pressure variability compared with propofol. These hemodynamic advantages translated into faster emergence and extubation, shorter hospital stays, and a significant treatment × surgical type interaction for extubation time, with the greatest benefit observed in patients undergoing abdominal procedures.

### Mechanistic considerations

The pronounced hemodynamic advantage of fospropofol is consistent with its unique pharmacokinetic profile. Unlike propofol, which produces a rapid peak plasma concentration followed by a steep decline, fospropofol undergoes enzymatic conversion by alkaline phosphatase in a rate-limited manner (8, 13). This creates a gradual increase in plasma propofol concentration that effectively functions as an endogenous target-controlled infusion. The resulting smoother pharmacokinetic profile attenuates the abrupt vasodilation and myocardial depression characteristic of bolus propofol administration (9, 10, 14, 15). The magnitude of the observed effect is noteworthy: a Cohen's d of 1.87 for MAP variability indicates a very large effect, suggesting that the pharmacokinetic advantage of fospropofol is particularly relevant in the pre-incision phase. The fact that heart rate variability did not differ between groups (*p* = 0.051) while SBP variability was markedly reduced supports the interpretation that fospropofol's benefit is mediated primarily through attenuation of vascular depression rather than chronotropic effects. Importantly, under a standardised BIS-guided protocol (target 40–60), the comparable anesthetic depth between groups — as evidenced by equivalent sevoflurane MAC values (1.00 vs. 1.01; *p* = 0.217), identical opioid dosing (*p* = 0.865), and similar muscle relaxant exposure (*p* = 0.995) — suggests that the hemodynamic advantages of fospropofol are not attributable to lighter anesthesia. Rather, the slower release kinetics of fospropofol achieve comparable anesthetic depth with a lower propofol-equivalent plasma concentration, which is precisely the mechanism underlying its hemodynamic advantage.

Maintenance dose and recovery time. A critical consideration is whether the observed recovery time advantage of fospropofol reflects pharmacological superiority or simply lower total anaesthetic exposure. The FOS group received a median maintenance dose of 5.0 mg/kg/h, which, applying the established conversion factor of approximately 0.54 mg propofol per mg fospropofol, yields a propofol-equivalent infusion rate of approximately 2.7 mg/kg/h, compared with 7.0 mg/kg/h in the PRO group. Importantly, within-group correlation analyses revealed no significant association between maintenance dose and emergence time (PRO: r = 0.019, *p* = 0.887; FOS: r = −0.012, *p* = 0.925) or extubation time (PRO: r = 0.051, *p* = 0.700; FOS: r = −0.042, *p* = 0.750), indicating that the recovery advantage of fospropofol is not explained by dose-dependent pharmacokinetic effects alone. This suggests that the gradual, enzyme-mediated release of propofol from fospropofol confers a genuine pharmacokinetic advantage beyond simple dose reduction (13). Furthermore, under BIS-guided titration, sevoflurane MAC, opioid dose, and muscle relaxant dose were equivalent between groups, confirming that the lower propofol-equivalent exposure in the FOS group did not result in lighter anesthesia. Had the FOS group been under-anesthetised despite BIS guidance, one would expect compensatory increases in sevoflurane requirements, reduced opioid or muscle relaxant dosing, or both; the absence of such differences argues firmly against the underdosing hypothesis.

### Surgical type interaction

The significant treatment × surgical type interaction for extubation time (*p* = 0.042) represents a novel finding with potential clinical implications. Abdominal surgery patients experienced a 3.58-minute reduction in extubation time with FOS compared with only 1.07 min in non-abdominal procedures. Several mechanisms may explain this differential effect. Abdominal surgery, particularly under laparoscopic conditions, imposes additional hemodynamic stress through pneumoperitoneum, Trendelenburg positioning, and visceral traction (16–18). Patients who maintain better hemodynamic stability during the pre-incision phase may enter the surgical phase with more favorable physiological reserve, leading to faster pharmacological clearance and recovery.

### Clinical implications

The findings have several implications for clinical practice. First, the NNT of 2.4 for hypotension indicates that propofol use in high-BMI patients carries a substantial risk of hemodynamic instability. Given the established association between intraoperative hypotension and end-organ injury (4, 5), the hemodynamic protection afforded by fospropofol may have implications beyond immediate perioperative outcomes. Second, the faster emergence and extubation times (Cohen's d 0.86–0.91) represent large effect sizes with operational relevance for surgical suite efficiency. Third, the shorter hospital length of stay in the FOS group (2.7 vs. 3.2 days; *p* = 0.011), while statistically significant, should be interpreted cautiously. Hospital discharge is influenced by multiple factors beyond anesthetic choice, including surgical factors, institutional discharge criteria, and postoperative complications. Although the multivariable analysis adjusting for surgical type and baseline characteristics suggests an independent association, a causal relationship cannot be established from this single-center trial.

### Comparison with previous literature

Previous studies comparing fospropofol with propofol have reported mixed hemodynamic findings (19). In a dose-finding study, fospropofol produced similar hemodynamic effects compared with propofol in general surgical patients without BMI stratification (20, 21). A study demonstrated more stable bispectral index profiles with fospropofol but did not report detailed hemodynamic outcomes (22). The present analysis extends these findings by focusing specifically on a high-BMI population with detailed pre-incision-phase monitoring, revealing a large hemodynamic advantage not previously documented. An important safety consideration specific to fospropofol is its metabolic pathway. Fospropofol is converted by alkaline phosphatase to release propofol, phosphate, and formaldehyde (8). Although formaldehyde is rapidly metabolised to formate by aldehyde dehydrogenase and further to carbon dioxide, concerns have been raised about potential accumulation with repeated or prolonged dosing. In the present study, no adverse events attributable to formaldehyde were observed, and all hepatic and renal function markers remained within clinically acceptable ranges at 24-hour follow-up. Previous phase 3 trials have similarly demonstrated acceptable safety profiles at standard clinical doses (21). Nevertheless, monitoring of formaldehyde-related toxicity may be warranted in patients receiving prolonged fospropofol infusions or those with impaired aldehyde dehydrogenase activity.

### Limitations

Several limitations merit consideration. First, the trial was originally designed and powered for metabolic rather than hemodynamic endpoints. The present report constitutes a pre-specified secondary analysis of hemodynamic outcomes. While the sample size was determined based on the metabolic primary endpoint, *post-hoc* power analysis confirmed that 120 patients provided 99.7% power for the observed 42-percentage-point difference in hypotension incidence (Cohen's h = 0.86). The randomised design preserves internal validity, and the observed effect sizes were robust across sensitivity analyses and multivariable adjustment. Nevertheless, readers should interpret the hemodynamic findings within the context of a secondary analysis framework. Second, the single-blind design introduces potential performance bias, particularly for subjective endpoints such as emergence and extubation times, where the unblinded anaesthesiologist's judgment and practice patterns may have influenced timing assessments. Third, the pre-incision-phase monitoring was limited to approximately 0–26 min, and findings cannot be extrapolated to maintenance or emergence periods. Fourth, although ASA class was included in the multivariable model, the modest imbalance in ASA III distribution (FOS 10.0% vs. PRO 20.0%) may have introduced residual confounding. Fifth, the study was conducted at a single center with a Chinese patient population, potentially limiting generalisability. Sixth, intraoperative hemodynamic management was at the discretion of the treating anesthesiologist. Finally, the surgical type interaction, while statistically significant, requires confirmation in prospective studies specifically powered for subgroup analyses. Although BIS was used for real-time clinical guidance during the procedure, BIS values were not systematically recorded in the case report form for *post hoc* analysis. Consequently, anesthesia depth equivalence is supported by proxy indicators (sevoflurane MAC, opioid dose, and muscle relaxant exposure) rather than direct BIS trajectory data. Future studies should incorporate continuous BIS recording to provide more granular evidence of depth equivalence.

## Conclusions

In high-BMI patients, fospropofol demonstrated markedly superior hemodynamic stability during the pre-incision phase compared with propofol, with a 42-percentage-point absolute reduction in hypotension incidence. This hemodynamic resilience translated into clinically meaningful improvements in emergence time, extubation time, and hospital length of stay. The significant treatment × surgical type interaction for extubation time suggests that patients undergoing abdominal surgery derive particular benefit. These findings support the consideration of fospropofol as a preferred induction agent in patients with elevated BMI, particularly those scheduled for abdominal surgical procedures. Prospective trials specifically designed and powered for hemodynamic endpoints are warranted to confirm these findings. These conclusions should be considered in light of the single-center design, the single-blind methodology with an unblinded attending anaesthesiologist, and the modest imbalance in ASA III distribution between groups.

## Data Availability

The original contributions presented in the study are included in the article/Supplementary Material, further inquiries can be directed to the corresponding author.

## Supplementary material

Supplementary Table S1 Subgroup analysis: hypotension incidence by BMI category, hypertension, and diabetes status.
