# Supplementary material for: Hemodynamic resilience and enhanced early recovery with fospropofol versus propofol in high-BMI patients undergoing surgery: a randomised controlled trial
Source: Front Surg. 2026 Jul 31;13:1877622. doi: 10.3389/fsurg.2026.1877622 (PMC13472999; doi:10.3389/fsurg.2026.1877622)
Supplement: Supplementary file 1 [file Datasheet1.pdf]

Supplementary Material 1

Trial Protocol Excerpt, Statistical Analysis Plan,
and Post-hoc Power Calculation for Hemodynamic Secondary Analysis

Accompanying: Hemodynamic Resilience and Enhanced Early Recovery with Fospropofol versus Propofol in High-BMI Patients

Trial Registration: ChiCTR2500101725

Section 1: Original Trial Protocol — Relevant Excerpts

The following excerpts are taken from the original trial protocol registered with the Chinese Clinical Trial Registry (ChiCTR2500101725, registered 29 April 2025). The complete protocol is available upon request from the corresponding author.

1.1 Study Title

Metabolic advantages of fospropofol disodium in patients with high BMI: a randomised, double-blind, controlled trial assessing short-term metabolic endpoints.

1.2 Study Design

Single-centre, prospective, randomised, double-blind, controlled clinical trial.

1.3 Objectives

Primary objective: To compare the effect of fospropofol versus propofol on alkaline phosphatase (ALP) change from baseline to 24 hours postoperatively in patients with BMI ≥ 28 kg/m².

Secondary objectives:

- To compare changes in other metabolic biomarkers (TC, TG, HDL-C, LDL-C) between groups.
- To compare intraoperative hemodynamic stability (MAP variability, hypotension incidence, vasopressor requirements) between groups.
- To compare early recovery profiles (emergence time, extubation time, hospital length of stay) between groups.
- To compare the incidence of adverse events (injection pain, hypertriglyceridaemia, PONV) between groups.

1.4 Primary Endpoint (Original Trial)

ALP change from baseline (T0) to 24 hours postoperatively (T2).

1.5 Pre-specified Secondary Endpoints

- Changes in TC, TG, HDL-C, and LDL-C from T0 to T2.
- Intraoperative MAP variability coefficient.
- Incidence of intraoperative hypotension (any MAP < 65 mmHg during the pre-incision phase).
- Emergence time (cessation of anaesthesia to eye opening on command).
- Extubation time (cessation of anaesthesia to successful extubation).
- Hospital length of stay (days).
- Incidence of injection pain, hypertriglyceridaemia (TG > 2.3 mmol/L at 24 h), and PONV (0–24 h).
- Vasopressor usage (type, incidence, cumulative dose).

Note: The complete list of secondary endpoints listed above reflects the original trial protocol. The first manuscript (metabolic endpoints) reported a subset relevant to metabolic outcomes; the present manuscript reports the hemodynamic and recovery subset.

1.6 Sample Size Calculation

The sample size was calculated for the primary metabolic endpoint (ALP change). Based on pilot data, an expected between-group difference of 12.5 U/L (SD 25 U/L) was assumed. With $\alpha = 0.05$ (two-sided) and 80% power, 54 participants per group were required. The sample size was inflated to 60 per group (total $N = 120$) to allow for a 10% attrition rate.

Section 2: Statistical Analysis Plan for Hemodynamic Secondary Analysis

This statistical analysis plan (SAP) describes the analytical approach for the hemodynamic and early recovery secondary outcomes of trial ChiCTR2500101725. The primary report of this analysis has been submitted as a separate manuscript.

2.1 Analysis Population

All randomised patients who received the allocated intervention and completed the study protocol were included in the analysis (intention-to-treat population). No patients were lost to follow-up (120/120 completed all three timepoints).

2.2 Primary Endpoint of This Secondary Analysis

The primary endpoint of this secondary analysis is the incidence of hypotension (any MAP < 65 mmHg) during the pre-incision phase, compared between the FOS and PRO groups.

2.3 Secondary Endpoints

- MAP variability coefficient during the pre-incision phase.
- MAP nadir during the pre-incision phase.
- Time-weighted average MAP < 65 mmHg (hypotension burden).
- Emergence time and extubation time.
- Vasopressor usage (incidence and cumulative dose).
- Anesthetic depth indicators (sevoflurane MAC, opioid dose, muscle relaxant dose).
- Fluid management (crystalloid, colloid, blood loss).
- Hospital length of stay.
- Treatment-by-surgical-type interaction for hemodynamic outcomes.

2.4 Statistical Methods

Continuous variables were reported as mean ± SD or median (IQR) depending on distribution normality (assessed by Shapiro–Wilk test). Between-group comparisons used independent-samples t-tests (normally distributed) or Mann–Whitney U tests (non-normal).

Categorical variables were reported as n (%) and compared using chi-squared or Fisher's exact tests as appropriate. Risk ratio (RR), absolute risk reduction (ARR), and number needed to treat (NNT) with 95% confidence intervals were calculated for the primary endpoint.

Logistic regression was used for multivariable adjustment of the primary endpoint, including treatment group, surgical type, baseline MAP, BMI, and ASA physical status as covariates.

Linear mixed-effects models were used for longitudinal MAP analysis, with random intercepts for patients and fixed effects for treatment group, time, and their interaction.

Treatment-by-subgroup interactions were assessed by adding interaction terms to the logistic regression model. Subgroups were defined by BMI stratum (28–32 vs. >32 kg/m²) and surgical type (abdominal vs. non-abdominal).

Sensitivity analyses included: (1) restriction to the first 15 minutes of monitoring; (2) ANCOVA adjusting for baseline MAP and BMI; (3) alternative hypotension definition (MAP decline >30% from baseline); and (4) Holm-Bonferroni correction for multiple comparisons.

All tests were two-sided with $\alpha = 0.05$. No multiplicity adjustment was applied for secondary endpoints, consistent with the exploratory nature of secondary analyses. Analyses were performed using Python 3.12 with scipy 1.14, statsmodels 0.14, and lifelines 0.29.

Section 3: Post-hoc Power Calculation

3.1 Rationale

The original trial was designed and powered for the metabolic primary endpoint (ALP change at 24 hours). As the present manuscript reports pre-specified secondary hemodynamic outcomes, a post-hoc power calculation was performed to determine the statistical power available for the observed between-group difference in hypotension incidence.

3.2 Method

Post-hoc power was computed for a two-proportion z-test using the observed event rates in each group. The calculation was verified using the statsmodels library in Python 3.12 (function: statsmodels.stats.power.zt_ind_solve_power) and independently confirmed with Cohen's h effect size formula.

3.3 Input Parameters

Parameter	Value
**Sample size per group**	n = 60
**Total sample size**	N = 120
**Observed hypotension rate (FOS)**	30.0% (18/60)
**Observed hypotension rate (PRO)**	71.7% (43/60)
**Observed between-group difference**	41.7 percentage points
**Cohen's h (effect size)**	0.860
**Significance level (α)**	0.05 (two-sided)

3.4 Calculation Details

Cohen's h was computed as:

$$h = 2 \times \arcsin(\sqrt{p_1}) - 2 \times \arcsin(\sqrt{p_2})$$

where p_1 = 0.300 (FOS hypotension rate) and p_2 = 0.717 (PRO hypotension rate).

$$h = 2 \times \arcsin(\sqrt{0.300}) - 2 \times \arcsin(\sqrt{0.717}) = 0.860$$

Using n = 60 per group and α = 0.05 (two-sided), the achieved power was:

Power = 99.7%

3.5 Verification Code (Python)

```
import numpy as np
from statsmodels.stats.power import zt_ind_solve_power
from statsmodels.stats.proportion import proportions_ztest

# Observed data
fos_rate = 18 / 60   # 0.300
pro_rate = 43 / 60   # 0.717

# Cohen's h
h = 2 * (np.arcsin(np.sqrt(fos_rate)) - np.arcsin(np.sqrt(pro_rate)))
print(f"Cohen's h = {abs(h):.3f}")
```

```
# Post-hoc power
power = zt_ind_solve_power(
    effect_size=abs(h),
    nobs1=60,
    alpha=0.05,
    ratio=1.0,
    alternative='two-sided'
)
print(f"Power = {power:.1%}")  # Output: 99.7%
```

3.6 Interpretation

The observed effect size (Cohen's h = 0.860) is classified as a large effect (Cohen's convention: $h > 0.80$ = large). With 60 patients per group, the achieved power of 99.7% far exceeds the conventional threshold of 80%. This indicates that the sample size, although determined for a different primary endpoint, provides more than adequate statistical power for the hemodynamic comparison.

The minimum detectable effect size at 80% power with $n = 60$ per group and $\alpha = 0.05$ (two-sided) would be Cohen's $h \approx 0.51$, corresponding to an approximate between-group difference of 25 percentage points — substantially smaller than the observed 41.7-percentage-point difference.

Section 4: Original Sample Size Derivation (Metabolic Primary Endpoint)

For transparency, the original sample size calculation for the metabolic primary endpoint is reproduced here. This calculation was performed during trial design and registered with the Chinese Clinical Trial Registry (ChiCTR2500101725).

4.1 Parameters

Parameter	Value
**Primary endpoint**	ALP change from baseline to 24 h (U/L)
**Expected between-group difference**	12.5 U/L
**Expected standard deviation**	25 U/L
**Significance level (α)**	0.05 (two-sided)
**Statistical power**	80%
**Required sample size per group**	54

4.2 Calculation

Using a two-sample t-test framework:

1. Effect size $d = \Delta/\sigma = 12.5/25 = 0.50$ (medium effect by Cohen's convention)

2. Standard two-sample formula: $n = 2 \times [(z_{0.975} + z_{0.80})/d]^2 = 2 \times [(1.96 + 0.842)/0.50]^2 \approx 63$ per group

3. The original protocol reports n = 54 per group, likely derived using ANCOVA-adjusted sample size (accounting for baseline covariate correlation) or software-specific corrections (e.g., G*Power with t-distribution approximation). The discrepancy between the standard formula (63) and the protocol value (54) reflects the different statistical frameworks used.

4. Inflated by 10% for potential attrition: $54 \times 1.10 \approx 59.4 \rightarrow 60$ per group

5. Total: N = 120

Note: The actual attrition rate was 0% (120/120 completed all follow-ups), providing the full planned sample for analysis.
